# Psychiatric emergencies: epidemiological analysis and healthcare professionals’ experiences

**DOI:** 10.1186/s12873-025-01268-y

**Published:** 2025-07-01

**Authors:** Selim Degirmenci, Nese Mercan

**Affiliations:** 1 Department of Emergency Medicine, Faculty of Medicine, Bilecik Seyh Edebali University, Bilecik, Türkiye; 2 Psychiatric Nursing Department, Faculty of Health Sciences, Bilecik Seyh Edebali University, Bilecik, Türkiye

**Keywords:** Agitation, Emergency medicine, Mental disorders, Phenomenology, Quality improvement

## Abstract

**Background:**

Emergency departments ensure the safety of patients with mental health disorders and initiate psychiatric treatment. However, emergency workers experience several challenges in approaching patients with mental health disorders. It is essential to describe the epidemiological characteristics of cases and determine healthcare workers’ opinions to identify the conditions that affect interventions. This study aimed to examine the epidemiology of patients evaluated for psychiatric emergencies in the emergency department and reveal healthcare professionals’ experiences with emergency psychiatric cases.

**Methods:**

The study comprised two parts. First, patients who attended Bilecik Training and Research Hospital’s Emergency Department with psychiatric emergencies between January 1, 2022, and December 31, 2023, were retrospectively examined. Second, using a phenomenological design and qualitative methodology, healthcare professionals were interviewed about their experiences and opinions regarding approaching patients who attended the emergency department with psychiatric emergencies and their diagnosis, treatment, and outcome processes.

**Results:**

In the first part of the study, 1621 patients were evaluated during the study period: 163 were aged < 18 years and 1458 were aged ≥ 18 years, while 1079 were female (67.0%). In terms of diagnoses, 1417 (87.4%) were evaluated for anxiety and 74 (4.6%) for suicide attempts. In the second part, four themes and 11 categories emerged from the analysis of the interviews conducted with healthcare professionals. These themes were the path from fear to stigmatization, the necessity of therapeutic relationships, the necessity of change in intervention conditions, and the factors that change the meaning of the experience.

**Conclusions:**

This study stresses the importance of considering healthcare professionals’ experiences and highlights the necessity for change in the emergency department. It is anticipated that fulfilling these needs will reduce acute distress and improve the safety and recovery of patients presenting with psychiatric emergencies.

## Background

Alongside those with physical symptoms, patients with symptoms of mental disorders also visit the emergency department (ED). The evaluations that are performed in the ED are vital and represent a crucial opportunity for diagnosis and treating these patients [1]. An examination of the reasons for attending the ED reveals that a significant portion of cases are psychiatric. In the United States, 8.3% of ED visits between 2007 and 2016 were due to psychiatric conditions or substance abuse; this figure rose from 6.6% in 2007 to 10.9% in 2016 [2]. A meta-analysis determined that patients presenting with psychiatric symptoms accounted for 4% of ED patients, 41.9% of whom had no prior psychiatric diagnosis. Therefore, the ED holds an important place in the care pathway for individuals who receive their first psychiatric diagnosis there. However, the same meta-analysis also revealed that 36% of patients diagnosed with a psychiatric condition were sent home directly from the ED; as such, the opportunity to provide mental health care may have been missed [3]. According to an Australian Institute of Health and Welfare report, the proportion of the Australian population who were receiving Medicare-subsidized mental health-specific services rose from 5.7% in 2008–2009 to 10.2% in 2017–2018. Additionally, when ED patients were examined nationwide, the proportion of patients who presented with symptoms of psychiatric disorders increased from 3.3% in 2013–2014 to 3.6% in 2017–2018 [4].

Acute psychiatric conditions that impact an individual’s thought processes and behaviors and impact their functionality are among the reasons for attending the ED. Individuals may experience a crisis when their coping mechanisms are inadequate and their perceived conditions are unrealistic; thus, they attend the ED [5]. Most individuals presenting to the ED with acute psychiatric conditions exhibit excitation, agitation, and self-harm behaviors. Individuals may also present to the ED when experiencing psychiatric symptoms, intoxication, substance abuse, the side effects of drugs, and drug-drug interactions [1]. Since these situations can result in fatal outcomes, the intervention implemented is crucial. In all such cases, the ED is where the individual’s safety is ensured and psychiatric treatment is initiated. Individuals who present to the ED with psychiatric symptoms are evaluated, differentially diagnosed, and—when necessary—pharmacotherapeutic, medical, and/or surgical treatment is planned, and practices, such as psychotherapy or social interventions, are implemented.

All interventions that are implemented in the ED are conducted in a limited time, with effective measures and rapid, correct guidance. Therefore, such interventions allow individuals to quickly resolve their issues and return to their lives without loss. Individuals who visit the ED with psychiatric symptoms experience a crucial journey from admission to outcome, a journey that is considered to be the only available option. However, the ED setting is often unsuitable for treating mental health needs [6]. Moreover, healthcare professionals may hold negative attitudes and stigma toward patients with psychiatric emergencies [7]. Such situations may adversely impact the desired intervention in emergency psychiatric cases [8]. Describing the epidemiological characteristics of cases and determining professionals’ opinions are, therefore, considered important in determining the conditions that affect interventions.

In Turkey, psychiatric patients can apply to three different groups of hospitals. The first consists of psychiatric hospitals, which solely provide care to mental health patients in all areas, including the ED. The second comprises hospitals that have a psychiatric inpatient unit among their wards, offer care to all patient groups, and most often do not employ healthcare professionals who specialize in psychiatry in the ED. The final group includes hospitals that do not have a psychiatric inpatient unit and their EDs are similar to the second group. Individuals with psychiatric cases who apply to this hospital and require hospitalization must be referred to another hospital with a psychiatric inpatient unit. Hospitals in the last two groups—particularly the final group—are considered to have limited resources to manage psychiatric cases in their EDs. It is unclear how this current situation regarding psychiatric case management impacts healthcare professionals and patients. To our knowledge, few studies have explored psychiatric emergency cases in Turkey, and none have qualitatively evaluated the opinions of healthcare professionals working in the ED in managing such cases. The lack of literature presents difficulties in describing experiences with psychiatric cases in the ED and prevents the identification of situations that require revision in practice. Thus, this study aims to determine whether ED practices in Turkey require revision and identify the changes required, thereby contributing data to support decisions on improving the quality of the interventions implemented by healthcare professionals in the ED. Moreover, it is anticipated that this may contribute to increasing awareness of emergency psychiatric cases and offer a foundation for future research in the area. The aim of the study is to conduct an epidemiological analysis of patients presenting to the ED with psychiatric emergencies and examine healthcare professionals’ experiences regarding such cases.

## Methods

### Study design

This study comprised two parts. The first part consisted of retrospectively examining patients who attended Bilecik Training and Research Hospital’s ED for psychiatric emergencies between January 1, 2022, and December 31, 2023. The second part comprised interviews with healthcare professionals involved in managing psychiatric emergency cases; their responses were analyzed via a phenomenological design and qualitative methodology.

### Study sample for the second part

Purposeful sampling was used to determine the study group; this method is a non-probability, non-random sampling approach that is preferred when studying one or more special cases that meet certain criteria or have certain characteristics [9]. To avoid bias, the researchers made joint decisions throughout the sample selection process and considered the diversity of healthcare professionals in terms of age, seniority, and shift. The study sample consisted of seven physicians, seven nurses, and seven paramedics, who were selected via a simple random sampling method from the 38 physicians and 39 nurses working in the hospital’s ED and the 46 paramedics working for the Central Provincial Ambulance Service of Bilecik. For the qualitative part, the inclusion criteria were volunteering for the study and having worked in the ED or ambulance service (AS) for at least a year. Healthcare professionals who did not treat or care for psychiatric emergency cases during the period of interest were excluded from the study.

### Data collection

The study retrospectively examined individuals who attended the ED of Bilecik Training and Research Hospital for psychiatric emergencies between January 1, 2022 and December 31, 2023. The diagnostic guidelines provided in the International Classification of Disease, tenth edition (ICD-10) were used to classify psychiatric disorder diagnoses. Patient demographic characteristics, including age, sex, month and season of admission, and diagnosis, were obtained via hospital records and patient files. For patients who attended more than once, their final admission was considered.

Qualitative data were obtained via individual semi-structured interviews with healthcare professionals until data saturation was achieved. This approach enabled participants to speak freely and offered deeper understanding and meaning [10] as participants could reveal the aspects and experiences that were most important to them regarding the study topic. The interviews were conducted conversationally, encouraging participants to be as open as possible about their thoughts and experiences. Thus, an attempt was made to access all of the participants’ experiences, making it possible to determine the nature or basis of the study topic. Each interview lasted approximately 45 min and was conducted face-to-face by NM, an expert in psychiatric nursing with experience in qualitative interviews. The interviews were conducted in a room in the ED that offered privacy and a healthy environment regarding temperature and noise. Following each interview, the researchers evaluated whether the study’s purpose had been fulfilled. The researchers decided data saturation had been achieved when the data became repetitive, new data did not offer a different perspective on the topic, and no new categories emerged. Each semi-structured question was asked fully to each participant to elicit their experiences and opinions. With the participants’ written and verbal consent, the interview was audio-recorded; the recordings were then transcribed verbatim and anonymized. The researchers gained familiarity with the contents of the recordings by listening to them several times. Notes were taken after each interview, which were used to highlight relevant information, such as body language.

### Tools

The researchers created and employed two forms for the study, in line with current developments on the basis of a literature review and expert opinion.

#### Healthcare professionals’ data collection form

This form comprised 12 questions, which collected data on the participants’ characteristics, including age, sex, profession, education status, duration of working in the ED or with the AS, and qualifications.

#### Semi-structured interview form

This form consisted of five questions evaluating healthcare professionals’ experiences of patients they managed in the ED (asked to doctors and nurses) or brought to the ED (paramedics) for psychiatric emergencies (Table 1).

**Table 1:** The semi-structured interview form

Questions
1. Could you describe your experiences with managing psychiatric emergency cases?
2. How has your experience with psychiatric emergency cases evolved over time, and what changes do you anticipate in the future?
3. What challenges and experiences do you encounter when managing patients at risk of self-harm or harm to others?
4. In what ways does the patient’s age influence your approach and experience in handling psychiatric emergencies?
5. What recommendations do you have for enhancing the management of psychiatric emergencies by healthcare professionals in the ED?

### Study rigor

In qualitative studies, validity is defined as the findings being accurate or reasonable, the interpretations being credible, and the research revealing information in regard to the topic of interest [11]. This study followed the necessary steps to ensure the validity and reliability of the qualitative analysis [12, 13]. Two impartial colleagues comprehensively evaluated the methodology, findings, and overall strategy of the study.

In the reporting section of the study, the purposeful sampling method was applied, the description was detailed, and transferability was ensured by including quotations from the participants, which demonstrated the research questions’ suitability for the study purpose and ensured the findings were accurate. Furthermore, the researchers’ field observations contributed to the study’s validity. Following the data analysis, participant confirmation was obtained to determine the extent to which the statements reflected their thoughts, showing that consistency was achieved between the researcher’s external view and the participants’ internal view. Two experts examined the research process to check for insufficient coding, incorrect categorization, or themes that were incompatible with the research content and topic. The study topic overlaps with the researchers’ field of expertise, which is regarded as essential in ensuring reliability. The study also assessed the ability of the themes to produce similar results during each measurement, and the analysis was repeated one month later. As agreement was achieved in both measurements, the study’s reliability was demonstrated.

### Data analysis

Quantitative data were analyzed using SPSS for Windows (version 22.0; SPSS Inc. Chicago, IL, USA). Descriptive statistics were used to summarize patient characteristics. The Kolmogorov-Smirnov test was employed to examine normality; when descriptive statistics were stated for numerical variables, data that showed normal distribution were expressed as mean ± Standard deviation (SD), and data that did not show normal distribution were expressed as median (minimum-maximum). Categorical variables were reported as numbers (percentages). To examine the differences in sex distribution between groups, the Chi-Square Independence test was applied. Meanwhile, Levene’s test was utilized to assess the homogeneity of variances, and since the assumption of homogeneity was not met, a One-Way Analysis of Variance (ANOVA) was applied with the Games-Howell post hoc test to compare mean differences between groups. Across all analyses, the significance level was accepted as *p* < 0.05.

The qualitative data were analyzed through an inductive qualitative content analysis to create themes and categories within the scope of the study. The form of thematic analysis is data-driven and establishes a robust connection between the data and the themes [14]. The researchers systematically examined the whole dataset, affording full and equal attention to each data element. Codes were created by underlying common or recurring concepts and combining them into a common category; following this, compelling aspects of the data elements were identified, forming the basis of the themes. To simplify identification, distinct colors were specified in a Microsoft Word document under various themes. The document included the participants’ identifying marks to ensure transparency and provide evidence for auditing purposes. This approach enabled an in-depth analysis by demonstrating the interrelationship of emerging themes. The data were reanalyzed to determine whether they fit the theme. This process led to the development of meaningful themes.

### Ethical considerations

The study received ethical approval from Bilecik Seyh Edebali University’s Non-Interventional Clinical Research Ethics Committee (approval number 7/9, dated 12/27/2023). The participants were duly informed of the study’s purpose and methodology, were able to withdraw from the study at any point, and were assured that their data would remain confidential. The study was conducted in accordance with the Declaration of Helsinki.

## Results

### Epidemiological data

Between January 1, 2022, and December 31, 2023, 264,890 individuals attended the ED. Of this number, 2736 (1%) individuals were experiencing psychiatric emergencies; 1115 repeat visits were excluded from the study. Among the 1621 patients evaluated, 163 (10.1%) were children (aged < 18 years) and 1458 (89.9%) were adults (aged ≥ 18 years), 1079 (67.0%) were female, and 327 (20.2%) were brought to the ED by ambulance. The patients’ mean age was 34.3 years (SD: 16.8). An examination of the differences in seasons demonstrated that more patients attended the ED in the summer than in other seasons (Table 2).

**Table 2:** Descriptive characteristics of patients experiencing psychiatric emergencies

Characteristic*	Female (*n* = 1079)	Male (*n* = 542)	Total (*N* = 1621)
Age (years)	35.6 ± 17.3	31.8 ± 15.6	34.3 ± 16.8
Age groups			
0–17	121 (11.2%)	42 (7.7%)	163 (10.1%)
18–34	437 (40.5%)	311 (57.4%)	748 (46.1%)
35–49	299 (27.7%)	110 (20.3%)	409 (25.2%)
50–65	160 (14.8%)	53 (9.8%)	213 (13.1%)
66–80	42 (3.9%)	19 (3.5%)	61 (3.8%)
≥ 81	20 (1.9%)	7 (1.3%)	27 (1.7%)
Season			
Winter	258 (23.9%)	119 (22.0%)	377 (23.3%)
Spring	263 (24.4%)	124 (22.9%)	387 (23.9%)
Summer	310 (28.7%)	181 (33.4%)	491 (30.3%)
Autumn	248 (23.0%)	118 (21.8%)	366 (22.6%)
Arrival at the ED			
Walk in	875 (81.1%)	419 (77.3%)	1294 (79.8%)
Ambulance	204 (18.9%)	123 (22.7%)	327 (20.2%)

*Data reported as the number (%) or mean ± SD

With respect to diagnosis, 1417 (87.4%) patients attended the ED due to anxiety, 74 (4.6%) due to a suicide attempt, and 61 (3.8%) due to bipolar affective disorder. A significant between-group difference was identified when patients were compared in terms of age and gender according to their diagnoses (P values = 0.001 and 0.001, respectively). For instance, patients in the suicide attempt and self-harm groups were significantly younger than other patients (Table 3). In terms of sex, there was a significant difference only between anxiety disorder and suicide attempt patients, and female patients were predominant in both groups (P values = 0.001 and 0.01, respectively) (Table 3).

**Table 3:** Comparison of age and sex distributions across disease groups

Diagnosis	*N* (%)	Age*	Sex (*N*, %)		*P*
			Female	Male	
Anxiety disorder	1417 (87.4%)	34.8 ± 17^a^	971 (68.5%)	446 (31.5%)	0.001
Suicide attempt	74 (4.6%)	28.2 ± 15^bc^	48 (64.9%)	26 (35.1%)	0.01
Bipolar affective disorder	61 (3.8%)	36 ± 16.4^ab^	31 (50.8%)	30 (49.2%)	NS
Self-harm	31 (1.9%)	23.7 ± 13.8^c^	13 (41.9%)	18 (58.1%)	NS
Schizophrenia	24 (1.5%)	34.7 ± 10.5^ab^	10 (58.3%)	14 (41.7%)	NS
Others	14 (0.9%)	39.5 ± 16.2^ab^	6 (42.9%)	8 (57.1%)	NS

*Data reported as mean ± SD. Any groups with different superscripts indicate statistically significant differences (*P* < 0.05)

### Healthcare professionals’ characteristics

The median age of the interviewees was 29.3 (24–51) years, and 16 (76.2%) participants were female. With respect to education level, nine (42.9%) held a graduate degree, and eight (38.1%) held a bachelor’s degree. In regard to marital status, 11 (52.4%) were single. In terms of professional experience, the median duration in the profession was 4.8 (1.5–27) years, while the median duration in the ED or AS was 3.7 (1–23) years. Seven interviewees had up to two years of professional experience, and the longest professional experience was 27 years for a paramedic. Additionally, 16 (76.2%) interviewees enjoyed working in the ED or AS. With respect to mental health, two (9.5%) interviewees had a mental health disorder, four (19.0%) were receiving psychological support, and two (9.5%) had at least one close relative with a mental health disorder. Notably, only one interviewee had received psychiatric training alongside their undergraduate/associate degree.

### Qualitative analysis

Four themes and 11 categories emerged from the analysis of the healthcare professionals’ interviews. The four themes were “the path from fear to stigma,” “the necessity of the therapeutic relationship,” “the necessity of change in intervention conditions,” and “the factors that change the meaning of experience” (Table 4).

**Table 4:** Themes and categories

Theme	Categories
The path from fear to stigmatization	Aggression Substance use history Anger
The necessity of the therapeutic relationship	Suicide intervention Sense of inadequacy
The necessity of change in intervention conditions	Environment Referral process Access to a psychiatrist
Factors that change the meaning of experience	Malingering Patient age Professional experience

### Themes

#### The path from fear to stigma

This theme consisted of the categories of aggression, history of substance abuse, and anger. Participants stated that the most challenging cases were individuals who exhibited aggression. Such cases revealed a sense of fear; the presence or history of substance use particularly exacerbated the intensity and effect of this feeling of fear. Additionally, the source of aggression was not related to mental disorders but to life-related stressors, causing participants to experience anger.

*When I notice the patient’s anger*,* I hesitate to approach the patient. When I know that they have a psychiatric disorder*,* I do not feel angry but sad. I also feel tired from dealing with the patient.* (Nurse 7)

*Even if a patient does not have a psychiatric disorder and does not have an aggressive attitude*,* when I notice that they are a little angry*,* I try to approach more calmly than my normal approach. Because*,* after all*,* they are a patient*,* I try to think*,* ‘They came to us because they need something’ at first. However*,* if this situation continues despite my calm speech*,* taking it easy*,* and calming them down*,* I start to get angry too.* (Doctor 1)

*It is slightly different in psychiatric patients. Our approach to those whose aggression is related to their illness is not the same as our approach to a patient who has insight. We try to communicate with them slightly more and enable them to express themselves.* (Doctor 2)

*I am generally a little afraid to approach psychiatric patients. Because I am afraid that they might attack me. Frankly*,* it is easier to deal with other patients at this point.* (Paramedic 7)

*The patient’s diagnosis of schizophrenia is enough to make me nervous. In other words*,* I try not to close the door*,* not to make eye contact*,* and if possible*,* I request that someone be with me while intervening. I approach them cautiously because of my fear. Although I have that fear inside me*,* I try not to show it to them. A long time has passed since I received my education on this subject*,* and I have not encountered many cases during this period. This may be why.* (Nurse 3)

*Patients who are addicted to substances may present with withdrawal symptoms. They are very agitated*,* uncontrollable*,* or alcoholic. These are the patient groups that I have the most difficulty with. I always have a fear inside me; what if I can’t control it? What if it comes after me while I’m leaving? I’ve never experienced this before*,* but I’ve witnessed it.* (Nurse 4)

*I am having difficulty communicating with a patient who presents with aggression during a manic phase. I cannot help this patient. This situation I am experiencing makes me sad.* (Doctor 2)

#### The necessity of the therapeutic relationship

This theme comprised the following categories: suicide intervention, and a sense of inadequacy. Each category exhibited the necessity of the therapeutic relationship between healthcare professionals and patients. Participants believed that a therapeutic relationship should be established with patients who visited the ED as a result of a suicide attempt. Alongside a feeling of incompleteness due to the inability to establish therapeutic communication, participants expressed curiosity about patients’ outcomes. The inability to establish a therapeutic relationship with patients made participants feel that they could not do anything for them, causing them to feel sad and helpless. Additionally, recurring admissions of the same patient triggered such thoughts and caused a loss of motivation. However, participants did not feel helpless after intervening in cases such as myocardial infarction (MI). Therefore, they would opt to intervene in cases of MI if they had the right to choose for the patient. Moreover, participants who expressed a lack of knowledge and feelings of inadequacy reported that they wished for competence in the therapeutic relationship.

*It makes me feel comfortable seeing that I can communicate with the patient. If I can’t communicate*,* I get nervous.* (Nurse 7)

*Suicide cases make me very sad. I don’t know what to say. I just say things like*,* ‘Hold out your arm; I’ll take your blood pressure.’ I don’t use phrases such as ‘don’t do it again; it’s not worth it.’ I’m sure it won’t do them any good. However*,* I can’t do anything else.* (Nurse 4)

*I don’t have difficulty in cases in which I can communicate effectively. There was a child diagnosed with autism*,* and after inappropriate communication by the security guard*,* his mother became very angry and started yelling continuously. The child then became aggressive. I established effective communication with the mother and the child. Afterward*,* both of them calmed down*,* and the problem was solved.* (Nurse 4)

*We have not received detailed education on this issue and have not encountered many such cases. We mostly perform the treatments that have been ordered. We stay away from the patient anyway. Therefore*,* we have no experience in this field. I have been working for twenty-five years*,* but if you ask me about the approach to psychiatric emergency patients*,* I can only tell you about my small experiences.* (Nurse 1)

*For example*,* an aggressive or agitated patient attends the emergency department. At first*,* there is a prejudice against them. Typically*,* there should not be such an approach. First*,* you should make them feel that you are listening; you should be interested. You should act close to the patient. Then*,* they listen to you anyway. They see you as closer. If you can communicate honestly*,* the patient also understands their mistake.* (Nurse 2)

*I listen to the psychiatric patient well and take their anamnesis much more deeply*,* and I enjoy it. The patient relaxes as they express themselves. They feel better when they see that I understand them. They feel that they are an important person when they see that I am listening.* (Doctor 5)

*Therefore*,* hearing constant swearing causes me to drift away from the profession. While you are trying for their health*,* they are insulting you. Some patients have endless questions and requests. I don’t have enough time to communicate effectively with patients. Not being able to communicate effectively makes me more tired.* (Nurse 6)

*I try to do my best for psychiatric patients. However*,* I can’t find a definitive solution like I do with MI. I can’t go into it; I feel like I’m just glossing over it. Maybe I’m buying time. That makes me feel helpless* (Doctor 6)

*I often don’t know what to talk about or how to act in psychiatric patients. Because we haven’t received enough education anyway. This tires me out a lot. That’s why I prefer to go to a multiple trauma patient rather than a psychiatric patient. Because there’s only physical fatigue.* (Paramedic 2)

*You cannot force a substance addict patient to see a psychiatrist. These are not a patient group that you can refer directly to a psychiatrist. It is completely up to them. What we can do in emergency conditions is very limited. We can call it incompleteness.* (Doctor 2)

*I feel sorry for people who try to commit suicide. I know they can’t solve their problems completely. There’s nothing we can do. I just say that ‘committing suicide is not the solution.’ They thank me when they leave. However*,* the place they go to will not be good for them*,* and they will try to commit suicide again and come back.* (Nurse 6)

#### The necessity of change in intervention conditions

It was determined that certain issues required change to benefit patients attending the ED with psychiatric disorders in their medical histories during the intervention. Changes are required in the ED to enhance the referral process, improve access to psychiatrists, ensure patient privacy and security, and allocate time to patients. For patients who are planning referral to hospitals with inpatient psychiatric services, the prolonged referral process and waiting in an unsuitable environment cause treatment to be challenging. When specialist opinions are needed, the lack of a psychiatric clinic or the inability to access a psychiatrist can prolong the evaluation process. At the same time, unsuitable physical environments prevent therapeutic communication due to the lack of privacy. Participants were also concerned about the patient’s risk of harming themselves or others.

*So antisocial people are difficult*,* especially when they apply while drunk. For example*,* the person already has an antisocial personality. When they drink alcohol*,* it becomes more difficult to manage. Even security forces cannot control them. They create chaos. For example*,* there may be a different area of care for these patients without ever taking them to the ED.* (Nurse 2)

*It is difficult to manage and follow a patient who has been waiting for a referral in the ED for a long time. There is no door in the area where the patient is being treated*,* and it is a curtained area. You hand the patient over to security*,* but you cannot trust them. Because you have to deal with other things at that time*,* sometimes the patient gets up and walks away. It is a bit difficult to follow them.* (Nurse 5)

*We have a security problem. Not next to the nurse*,* but in front of the patient’s door*,* so there should be closer security. I think it would be better if they were in a place where they could notice the incident faster and intervene faster.* (Nurse 7)

*There are patients in the ED who are not well and require admission to a closed psychiatric ward. They tie me into knots even more*,* and I have a hard time. Even if there is no psychiatrist or psychiatric assistant in the ED*,* it would be nice to have a psychiatric clinic. A more competent person in the field can help me convince the patient and help me in cases where forced treatment is needed.* (Doctor 3)

*The biggest obstacle to an effective interview with a patient who has attempted or thought about suicide is the environment of the ED. The ED is not a place where this interview can be carried out healthily. There is constant patient turnover. I have to spend at least half an hour with a person who has suicidal thoughts. While you are attending to that patient*,* another patient with a life-threatening condition due to a traffic accident arrives. Which is the priority: mental distress or a life-threatening condition? You go for the one with a life-threatening condition.* (Doctor 4)

*It would be more logical for a psychiatrist to come and communicate with patients who have attempted or thought about suicide. We can be sufficient to a certain extent. I believe that a specialist should communicate with them. I think that psychiatrists should be on shift in the ED. In addition*,* there should be a separate area for psychiatric patients. When they are in the same area as other patients*,* the psychiatric patient can become agitated*,* or the other patients can misinterpret the situation.* (Nurse 3)

#### Factors that change the meaning of experience

Certain variables, including malingering, patient age, and the healthcare professional’s experience, made it possible to make sense of the distinction between cases presenting with the same symptoms. Participants reported feeling sadness if the harming event was a depression or life-related stressor, while they felt anger if it was due to malingering. In the same situation, if the patient was an adolescent, participants felt anger toward the patient’s family and sadness toward the patient; if the patient was an adult, they reported less sadness. When the effect of participants’ level of experience was examined, the anxiety and fear toward psychiatric events that many experienced in the first year became manageable with experience. However, an inadequacy in coping caused burnout and a loss of motivation. When participants’ responsibilities toward the same cases were explored, physicians assumed medical and legal responsibilities, nurses administered treatment and monitored the patient, and paramedics delivered first aid in the ambulance and during the most intense moments of crisis. The fact that the three groups differed in responsibilities but had similar duties caused distinct experiences of the same cases.

*I was not angry with the patient who kicked me. They wouldn’t have done it if they did not have a psychiatric disorder. Adolescents who are angry with their lovers or who cannot get their parents to do something pretend to faint. I get very angry with them.* (Paramedic 1)

*Of course*,* sometimes there is anger. Because if you are intervening in 10 cases with an ambulance in shifting and six of them are patients who have consumed alcohol*,* even if I do not show it externally*,* I can feel bad thoughts inside that I cannot recognize myself. It becomes very difficult to deal with these problems after a certain point. For example*,* when we go to the scene*,* sometimes the police have not arrived yet. They arrive much later than us. Sometimes we say*,* ‘Instead of waiting for the police*,* let’s take the patient in the ambulance and go to the hospital.* (Paramedic 2)

*I often feel sorry for patients who attempt suicide because I can see the pain they are going through. Some patients who attempt suicide make me angry. Sometimes*,* there are patients who make me think that they are not trying to commit suicide but rather coming to the hospital to attract attention.* (Nurse 7)

*For example*,* they took three Parol* [acetaminophen] *tablets. They know that nothing will happen*,* but they say*,* ‘I wanted to commit suicide.’ Here*,* they are tiring out both the ambulance team*,* the healthcare professionals in the ED*,* and their own relatives. If it is not a psychiatric disorder but just a conversion or attention-seeking reason*,* this situation makes me angry.* (Doctor 1)

*I try to help elderly patients with all situations*,* not just psychiatric cases. Normally*,* if a young patient says*,* ‘I’m lonely*,*’ I don’t care much*,* but when an elderly patient says this*,* I want to at least get a psychiatric opinion.* (Doctor 1)

*Suicide cases make me feel sad and helpless. When it happens to a young patient*,* it becomes even sadder.* (Doctor 2)

*We are three people in the back of the ambulance trying to mechanically restrain the patient. The area is very narrow. We are taking the patient to another city. A three-hour journey awaits us. And anything can happen. Many things are going through my mind. While I think that they could harm us*,* I also wonder if they had a traumatic experience in the past. I experience fear*,* sadness*,* and many other emotions.* (Paramedic 6)

*She had taken 14 pills and was crying*,* saying*,* ‘I don’t want my family to know.’ The patient was 18 years old*,* a small patient. She had come alone. I don’t know what to think*,* but I feel sorry for her. I wish young people weren’t in this situation. I feel even sadder for young people.* (Paramedic 7)

*I had a 15-year-old patient who attempted suicide. I felt very sorry for her. She is more dependent than an adult. If she wants to leave home*,* she cannot; she does not have her own freedom. Her family is the one who upsets her*,* but she does not have the strength to get away from her family. That’s why I am angry with the family.* (Doctor 6)

*We stay longer with alcohol addicts. Opening a vein*,* giving an injection*,* doing wound care… Our interventions take a long time. Inevitably*,* we are in considerable contact with the patient before*,* during*,* and after the procedure. That’s the only thing that bothers me.* (Nurse 5)

*It is important how much alcohol the patient who comes after drinking alcohol has consumed before applying. I will not be able to obtain information about how much they have drunk because they are not conscious. It is difficult to examine these patients because of their general condition. It is difficult to manage. Then*,* I get angry.* (Doctor 3)

*As the years go by and I gain experience*,* I learn. Five years ago*,* I was running away from an aggressive patient because I was afraid*,* but now I can handle it. Five years later*,* maybe I can communicate better and be less nervous. I think that my communication with that patient can be stronger because I will have encountered such situations more often.* (Nurse 7)

*I used to be more emotionally involved with patients. Now*,* I still have the same interest and intervention in psychiatric patients in the ED. However*,* I started to think systematically. I can calm patients down and then continue with my other work. I started to see psychiatric disorders as other organic disorders.* (Doctor 4)

## Discussion

Evaluating interventions for psychiatric cases in the ED is crucial for enhancing care standards. In this regard, the interventions undertaken in EDs must be thoroughly examined [15]. This study conducted an epidemiological analysis of emergency psychiatric cases encountered by healthcare professionals working in EDs during their care processes and evaluated healthcare professionals’ experiences regarding such cases.

The study determined that psychiatric patients presenting to the ED over the two-year period constituted 1% of all patients, and 69% of patients were female. In line with recent findings, a study by Kiyak et al. demonstrated that psychiatric patients presented 2.3% of ED admissions, of whom 69% were female [16]. In one study conducted in France, the mean age of psychiatric patients presenting to the ED was 35.9 ± 15.2—which was similar to recent studies—while 53.2% of the patients were female, indicating a more balanced sex distribution [17]. In a study conducted in Turkey, the presenting symptoms most common among 500 psychiatric patients admitted to the ED were psychotic disorders (68%), depressive symptoms (9.8%), and suicide attempts (7.6%) [16]. In the study from France, depressive symptoms were reported at 31.9%, anxiety at 30.4%, and suicide attempts at 6.3% [17]. The present study identified the most common diagnoses as anxiety (87.4%), suicide attempts (4.6%), and bipolar affective disorder (3.8%). The distinctions in these findings suggest that the reasons for applying to the ED can vary depending on the region in which the study was conducted. The present study determined that patients who applied for suicide attempts (28.2 ± 15) and self-harm (23.7 ± 13.8) were younger. Youth suicide is regarded as a significant public health concern while self-harming behaviors—including suicide—are a leading cause of death among adolescents aged 15–29 worldwide [18, 19]. This study determined that being female carries a greater risk for anxiety disorder; this is supported by studies with similar findings, which are explained by both biological and psychosocial factors [20, 21]. The study also found that being female is a risk factor for suicide attempts. While socio-cultural gender norms may explain the reason for more suicide attempts, other unmeasured factors also play a role [22]. To clarify this issue, studies with high evidence value and that focus on social, behavioral, and cognitive variables are required.

Many interventions are used within the ED, such as simple supportive and problem-solving methods, suicide prevention, psychiatric consultation, and decisions to discharge, refer, or admit the patient [1]. The process involves many healthcare professionals, from taking psychiatric emergency cases from their homes to discharging them from the hospital. The present study revealed the experiences of paramedics, nurses, and physicians regarding patients presenting with mental health disorders. Four themes emerged from the analysis of the healthcare professionals’ interviews: the path from fear to stigmatization, the necessity of the therapeutic relationship, the necessity of change in intervention conditions, and the factors that change the meaning of the experience.

### The path from fear to stigmatization

Angry patients in the ED always carry the risk of violence, and it is crucial that signs of impending violence are identified. While the cause of anger can be varied—including delusions, hallucinations, and substance-related or similar psychiatric symptoms—life-related stressors can also elicit anger in those who present to the ED. Approaches such as verbal calming techniques and physical or chemical restraint can be employed in the ED to intervene with aggression [23]. In this study, the interviewed healthcare professionals experienced fear throughout the process of recognizing and intervening with anger. Additionally, other sources of anger than psychiatric disorders caused anger in the interviewees. Angry patients with a history of substance abuse or the belief that they are under the influence of substances caused a rapid sense of fear among healthcare professionals. Even if individuals were not angry, a history of substance use caused healthcare professionals to feel that “they could cause harm,” leading to the stigmatization of patients. One study explored the experiences of individuals with a history of substance use and identified that they felt stigmatized by healthcare professionals in the ED [24]. The findings of the current study indicate that stigma can be prevented and that patients’ feelings of stigma can be eliminated if healthcare professionals know how to manage anger.

### The necessity of the therapeutic relationship

In the present study, participants reported being aware that the therapeutic approach was important when they intervened in emergency psychiatric cases and acknowledged that it benefited both the patient and the process. It has been reported that individuals who have attempted suicide are among those for whom therapeutic communication is required. Patients who visit the ED due to a suicide attempt should receive rapid, sensitive intervention, their medical requirements should be assessed, and the necessary treatment should be initiated promptly. They should also be met with empathy and support, professional assistance and evaluation, a safe environment, and support interventions throughout recovery [25]. In the present study, the interviewees stated that they could not provide security, the physical environment was unsuitable for conducting therapeutic interviews, they had insufficient information on approaching such cases, and they felt that their interview skills were inadequate to manage such cases. In addition, they believed that their therapeutic approach was deficient for patients who visited the ED following a suicide attempt, as they focused more on physical symptoms.

One study reported that, compared to healthcare professionals working in other clinics, those working in the ED were less likely to have close relationships, despite being amidst a crisis and caring for patients experiencing a high level of stress [26]. The barriers healthcare professionals experience when intervening in patients experiencing complex, emotional situations in the ED should be addressed. The desired support and assistance should be delivered with a human-centered approach. Regardless of the model applied, a supportive therapeutic relationship must be rapidly established in the ED. Correct decision-making can be achieved by establishing patient cooperation across the therapeutic relationship [8]. One study explored the effects of emergency nursing practitioners, stressing the necessity of continuity in emergency intervention-specific training to enhance the quality of treatment and care and increase nurses’ motivation over time [27].

### The necessity of change in intervention conditions

Based on their experiences with psychiatric cases, the healthcare professionals interviewed in our study stated that changes should be made to reduce the difficulties they experience and improve patient care. Specifically, they desired changes to the physical environment, referral chain, and security policies. Participants reported that the physical environment was unsuitable for ensuring patient safety and comfort during waiting periods for patients scheduled for referral; the environment did not offer patients sufficient privacy, which prevented therapeutic interviews. Patients meeting with a psychiatrist or waiting for a referral in this environment also created challenges for healthcare professionals. The waiting time for patients who are planning to be referred can sometimes be extended to two to three days. The ED environment could be overcrowded, stimulating, and restricted by time pressures, making it unsuitable for individuals presenting with psychological problems to receive support [28].

The global need and focal points for change in treating psychiatric emergencies have previously been highlighted [8]. Studies have stressed the importance of evaluating the suitability of the ED’s physical environment for all types of interventions, making the necessary revisions, and reevaluating revisions [29, 30]. In one study, the opinions of ED-based healthcare professionals emphasized the importance of management leadership, available resources, organization-wide communication, and network arrangements for enhancing patient satisfaction and quality [15]. Another study identified that the implementation of psychiatric ED models aligned with the recommendations could decrease psychiatric hospitalizations [31]. On the basis of their experiences, individuals with psychiatric disorders who visited the ED wished for similar changes in regard to management as healthcare professionals [6]. Thus, our findings are aligned with the existing literature and indicate the need for change in interventions for psychiatric patients in the ED.

### Factors that change the meaning of experience

In this context, the age and malingering status of the patient and the experience of the healthcare professional affected the attribution of meaning in similar situations. There is a decreased likelihood of a healthcare professional establishing a therapeutic relationship with a patient when the latter is aggressive, and the meaning attributed to the experience of the profession (paramedic) that establishes physical and emotional contact with the patient at the peak of the crisis changes. Our findings demonstrated that professionals can interpret the same event differently. Similarly, another study identified low similarity in the decisions made by different professionals when determining whether patients can make their own decisions [32]. Our study revealed that the level of distress experienced by healthcare professionals differed depending on whether the patient was an adolescent or an adult, even if they presented with similar stories. Therefore, individuals who visited the ED required interventions with age-specific models [8]. These age-specific models should integrate information about age-specific issues and their solutions into interventions.

Among patients presenting with similar symptoms, those with a previous diagnosis of a mental disorder can cause anxiety and depression in healthcare professionals, whilst those who present with malingering can cause anger. In the prior literature, malingering is reported to be common in EDs and is most frequently associated with suicidal ideation (58.0%) and depression (39.0%) [33, 34], presenting a challenging situation for healthcare professionals [35]. Thus, the anger experienced by the participants of our study may be related to the high likelihood of encountering malingering. When the effects of professional experience were explored, anxiety toward the intervention and fear arising from patients’ thoughts of harm were lower in the first years, although burnout and loss of motivation increased. Similarly to our study, one systematic study determined that certain variables, including age and experience, of healthcare professionals working in the ED affected their attitudes toward mental disorders [36]. A further study revealed that working conditions were a source of unhappiness among nurses working in the ED, with differences in unhappiness levels according to their years of experience [37].

The themes of this study offer implications for ED-based health professionals’ daily practices in relation to psychiatric cases. Firstly, when the theme of stigma was addressed, the feeling of fear or a lack of safety experienced during interventions in aggressive situations may cause difficulties in managing crisis situations in routine practice. Secondly, inadequacy in therapeutic communication can adversely affect both the approach to the patient and the quality of care in routine practice. Thirdly, the need for change in the ED indicates that current conditions restrict routine practice and suggests the desire for the implementation of the ideal intervention. Finally, the factors that affect healthcare professionals’ practices, including the patient’s age, malingering, and the professionals’ experience, should not be overlooked. These factors may reduce the healthcare professionals’ empathic approach, decrease communication quality, and create bias in clinical evaluation processes.

### Limitations

This study had several limitations. Firstly, it was conducted in a specific culture and geographic region, potentially restricting the generalizability of its findings to other cultures and countries. Secondly, the generalizability of the findings may be further limited by differences in ED environments and healthcare professionals’ training between Türkiye and other countries. The hospital where the study was conducted has neither an inpatient psychiatric ward nor a psychiatrist or psychiatric nurse on shift in the ED. The hospital is the only one in a small city, and emergency psychiatric cases are generally referred to comprehensive hospitals in surrounding provinces for treatment. These characteristics of the hospital should be considered in the generalization of the findings. Additionally, healthcare professionals with different qualifications in larger hospitals in Turkey or EDs in other countries may expand the variety of qualitative data. To obtain a more comprehensive perspective, future studies should examine a broader range of psychiatric cases and the healthcare professionals involved in treating them.

## Conclusions

This study revealed the experiences of emergency healthcare professionals who must urgently intervene in cases of individuals experiencing psychiatric emergencies who present to the ED after their coping mechanisms fail to work and they experience scattered, intolerable thoughts. The study determined that healthcare professionals experience a path from fear to stigmatization, which may adversely impact their approach to patients. Our results highlight the importance of developing strategies to reduce stigma and offer a safe environment. Furthermore, healthcare professionals expressed that establishing a therapeutic relationship with the patient should be implemented into routine practice. To ensure this, training programs should be planned to increase the competence of healthcare professionals, and their practices can be accompanied by experts in psychiatry. Another finding of the study is the necessity of change in intervention conditions. In this regard, policy changes should include modifications to the physical structure of the ED environment. For psychiatric cases, a specialized area can be created in the ED where therapeutic communication can be established and the patient’s safety can be ensured. Psychiatric ED models with high evidence value can be applied to facilitate patient recovery. Additionally, cases requiring referral to another hospital should be referred appropriately and the quality, outcomes, and efficiency of referral care should be monitored. The study achieved an awareness of the factors that change the meaning of experience. It is recommended that further studies focus on how the identified factors are reflected in practice and concentrate on determining additional factors.

In conclusion, the experiences of healthcare professionals are essential in reducing acute distress and ensuring the safety and recovery of patients presenting in the ED with psychological symptoms. Changes made in the ED environment based on experiences may help resolve issues and ensure the quality of care.

## Data Availability

The data of this study are available from the corresponding author upon reasonable request.

## Abbreviations

AS: Ambulance service
ED: Emergency department
ICD-10: International Classification of Disease, tenth edition
MI: Myocardial infarction
SD: Standard deviation

## References

[1] Shah H, Somaiya M, Chauhan N, Gautam A. Clinical practice guidelines for assessment and management of children and adolescents presenting with psychiatric emergencies. Indian J Psychiatry. 2023;65(2):159–74. 10.4103/indianjpsychiatry.indianjpsychiatry_494_22. 37063627 10.4103/indianjpsychiatry.indianjpsychiatry_494_22 PMC10096198

[2] Theriault KM, Rosenheck RA, Rhee TG. Increasing emergency department visits for mental health conditions in the united States. J Clin Psychiatry. 2020;81(5):20m13241. 10.4088/JCP.20m13241. 32726001 10.4088/JCP.20m13241

[3] Barratt H, Rojas-García A, Clarke K, Moore A, Whittington C, Stockton S, et al. Epidemiology of mental health attendances at emergency departments: systematic review and Meta-Analysis. PLoS ONE. 2016;11(4):e0154449. 10.1371/journal.pone.0154449. 27120350 10.1371/journal.pone.0154449 PMC4847792

[4] Australian Institute of Health and Welfare. 2019. Mental health services—in brief 2019. Cat. no. HSE 228. Canberra: AIHW. 10.25816/5ec5bac5ed175

[5] Kaltsidis G, Bamvita JM, Grenier G, Fleury MJ. Predictors of frequent emergency department utilization for mental health reasons. J Behav Health Serv Res. 2021;48(2):259–73. 10.1007/s11414-020-09695-4. 32185614 10.1007/s11414-020-09695-4

[6] Roennfeldt H, Wyder M, Byrne L, Hill N, Randall R, Hamilton B. Subjective experiences of mental health crisis care in emergency departments: A narrative review of the qualitative literature. Int J Environ Res Public Health. 2021;18(18):9650. 10.3390/ijerph18189650. 34574574 10.3390/ijerph18189650 PMC8471743

[7] Abdelmonaem YMM, Osman MA, Karim NAHA. Mental health stigma and internship nursing students’ attitudes toward seeking professional psychological help: a cross-sectional study. BMC Nurs. 2024;23(1):275. 10.1186/s12912-024-01910-3. 38658957 10.1186/s12912-024-01910-3 PMC11044461

[8] Johnson S, Dalton-Locke C, Baker J, Hanlon C, Salisbury TT, Fossey M, et al. Acute psychiatric care: approaches to increasing the range of services and improving access and quality of care. World Psychiatry. 2022;21(2):220–36. 10.1002/wps.20962. 35524608 10.1002/wps.20962 PMC9077627

[9] Luborsky MR, Rubinstein RL. Sampling in qualitative research: rationale, issues, and methods. Res Aging. 1995;17(1):89–113. 10.1177/0164027595171005. 22058580 10.1177/0164027595171005 PMC3207270

[10] Doody O, Noonan M. Preparing and conducting interviews to collect data. Nurse Res. 2013;20(5):28–32. 10.7748/nr2013.05.20.5.28.e327. 23687846 10.7748/nr2013.05.20.5.28.e327

[11] Cypress BS. Rigor or reliability and validity in qualitative research: perspectives, strategies, reconceptualization, and recommendations. Dimens Crit Care Nurs. 2017;36(4):253–63. 10.1097/DCC.0000000000000253. 28570380 10.1097/DCC.0000000000000253

[12] Ahmed I, Ishtiaq S. Reliability and validity: importance in medical research. J Pak Med Assoc. 2021;71(10):2401–6. 10.47391/JPMA.06-861. 34974579 10.47391/JPMA.06-861

[13] Sim J, Saunders B, Waterfield J, Kingstone T. Can sample size in qualitative research be determined a priori? Int J Soc Res Methodol. 2018;21(5):619–34. 10.1080/13645579.2018.1454643.

[14] Castleberry A, Nolen A. Thematic analysis of qualitative research data: is it as easy as it sounds? Curr Pharm Teach Learn. 2018;10(6):807–15. 10.1016/j.cptl.2018.03.019. 30025784 10.1016/j.cptl.2018.03.019

[15] Anaraki NR, Mukhopadhyay M, Jewer J, Patey C, Norman P, Hurley O, et al. A qualitative study of the barriers and facilitators impacting the implementation of a quality improvement program for emergency departments: surgecon. BMC Health Serv Res. 2024;24(1):855. 10.1186/s12913-024-11345-w. 39068432 10.1186/s12913-024-11345-w PMC11283688

[16] Kiyak R, Gizli G, Koksal O. Epidemiological investigation of patients admitted to the emergency department and considered as psychiatric emergencies. Balıkesir Sağlık Bilimleri Dergisi. 2024;13(3):566–71. 10.53424/balikesirsbd.1481436.

[17] Akkaoui MA, Barruel D, Dauriac-Le Masson V, Gourevitch R, Pham-Scottez A. Trends in psychiatric emergency visits: insights from france’s largest psychiatric emergency department. Int J Emerg Med. 2025;18(1):13. 10.1186/s12245-025-00810-w. 39815203 10.1186/s12245-025-00810-w PMC11734344

[18] Stelmach R, Kocher EL, Kataria I, Jackson-Morris AM, Saxena S, Nugent R. The global return on investment from preventing and treating adolescent mental disorders and suicide: a modelling study. BMJ Glob Health. 2022;7(6):e007759. 10.1136/bmjgh-2021-007759. 35705224 10.1136/bmjgh-2021-007759 PMC9240828

[19] Balt E, Mérelle S, Robinson J, Popma A, Creemers D, van den Brand I, et al. Social media use of adolescents who died by suicide: lessons from a psychological autopsy study. Child Adolesc Psychiatry Ment Health. 2023;17(1):48. 10.1186/s13034-023-00597-9. 37029395 10.1186/s13034-023-00597-9 PMC10082488

[20] Farhane-Medina NZ, Luque B, Tabernero C, Castillo-Mayén R. Factors associated with gender and sex differences in anxiety prevalence and comorbidity: A systematic review. Sci Prog. 2022;105(4):368504221135469. 10.1177/00368504221135469. 36373774 10.1177/00368504221135469 PMC10450496

[21] Kundakovic M, Rocks D. Sex hormone fluctuation and increased female risk for depression and anxiety disorders: from clinical evidence to molecular mechanisms. Front Neuroendocrinol. 2022;66:101010. 10.1016/j.yfrne.2022.101010. 35716803 10.1016/j.yfrne.2022.101010 PMC9715398

[22] Bommersbach TJ, Rosenheck RA, Petrakis IL, Rhee TG. Why are women more likely to attempt suicide than men? Analysis of lifetime suicide attempts among US adults in a nationally representative sample. J Affect Disord. 2022;311:157–64. 10.1016/j.jad.2022.05.096. 35598742 10.1016/j.jad.2022.05.096

[23] Wheat S, Dschida D, Talen MR. Psychiatric emergencies. Prim Care. 2016;43(2):341–54. 10.1016/j.pop.2016.01.009. 27262012 10.1016/j.pop.2016.01.009

[24] Hawk K, McCormack R, Edelman EJ, Coupet E Jr, Toledo N, Gauthier P, et al. Perspectives about emergency department care encounters among adults with opioid use disorder. JAMA Netw Open. 2022;5(1):e2144955. 10.1001/jamanetworkopen.2021.44955. 35076700 10.1001/jamanetworkopen.2021.44955 PMC8790663

[25] Betz ME, Boudreaux ED. Managing suicidal patients in the emergency department. Ann Emerg Med. 2016;67(2):276–82. 10.1016/j.annemergmed.2015.09.001. 26443554 10.1016/j.annemergmed.2015.09.001 PMC4724471

[26] Thomas B, McGillion A, Edvardsson K, O’Meara P, Van Vuuren J, Spelten E. Barriers, enablers, and opportunities for organisational follow-up of workplace violence from the perspective of emergency department nurses: a qualitative study. BMC Emerg Med. 2021;21(1):19. 10.1186/s12873-021-00413-7. 33579206 10.1186/s12873-021-00413-7 PMC7880205

[27] Lloyd-Rees J. How emergency nurse practitioners view their role within the emergency department: A qualitative study. Int Emerg Nurs. 2016;24:46–53. 10.1016/j.ienj.2015.06.002. 26163111 10.1016/j.ienj.2015.06.002

[28] Savioli G, Ceresa IF, Gri N, Bavestrello Piccini G, Longhitano Y, Zanza C, et al. Emergency department overcrowding: Understanding the factors to find corresponding solutions. J Pers Med. 2022;12(2):279. 10.3390/jpm12020279. 35207769 10.3390/jpm12020279 PMC8877301

[29] Kirk JW, Nilsen P, Andersen O, Stefánsdóttir NT, Lindstrøm MB, Powell BJ, et al. Creating a sense of place when implementing a new emergency department in denmark: a qualitative study. BMC Health Serv Res. 2024;24(1):1482. 10.1186/s12913-024-11980-3. 39604991 10.1186/s12913-024-11980-3 PMC11600571

[30] Stefánsdóttir NT, Nilsen P, Lindstroem MB, Andersen O, Powell BJ, Tjørnhøj-Thomsen T, et al. Implementing a new emergency department: a qualitative study of health professionals’ change responses and perceptions. BMC Health Serv Res. 2022;22(1):447. 10.1186/s12913-022-07805-w. 35382815 10.1186/s12913-022-07805-w PMC8985264

[31] Kim AK, Vakkalanka JP, Van Heukelom P, Tate J, Lee S. Emergency psychiatric assessment, treatment, and healing (EmPATH) unit decreases hospital admission for patients presenting with suicidal ideation in rural America. Acad Emerg Med. 2022;29(2):142–9. 10.1111/acem.14374. 34403550 10.1111/acem.14374 PMC8850530

[32] Sonkurt H, Altınöz Ş, Coşkun A, Altınöz A. Comparison of emergency department and psychiatry physicians’ views on decision-making capacity cases in the grey zone. Turkish J Clin Psychiatry. 2022;25(2):177–83. 10.5505/kpd.2022.13245.

[33] Rumschik SM, Appel JM. Malingering in the psychiatric emergency department: prevalence, predictors, and outcomes. Psychiatr Serv. 2019;70(2):115–22. 10.1176/appi.ps.201800140. 30526343 10.1176/appi.ps.201800140

[34] Simpson SA, Loh R, Goans CRR, Ryall K, Middleton M, Dalton A. Suicide and Self-Harm outcomes among psychiatric emergency service patients diagnosed as malingering. J Emerg Med. 2021;61(4):381–6. 10.1016/j.jemermed.2021.05.016. 34210531 10.1016/j.jemermed.2021.05.016

[35] Zubera A, Raza M, Holaday E, Aggarwal R. Screening for malingering in the emergency department. Acad Psychiatry. 2015;39(2):233–4. 10.1007/s40596-014-0253-1. 25476227 10.1007/s40596-014-0253-1

[36] Clarke D, Usick R, Sanderson A, Giles-Smith L, Baker J. Emergency department staff attitudes towards mental health consumers: a literature review and thematic content analysis. Int J Ment Health Nurs. 2014;23(3):273–84. 10.1111/inm.12040. 23980913 10.1111/inm.12040

[37] Javanmardnejad S, Bandari R, Heravi-Karimooi M, Rejeh N, Sharif Nia H, Montazeri A. Happiness, quality of working life, and job satisfaction among nurses working in emergency departments in Iran. Health Qual Life Outcomes. 2021;19(1):112. 10.1186/s12955-021-01755-3. 33794917 10.1186/s12955-021-01755-3 PMC8017644

